# Inhibition of endolysosomal two-pore channel 2 (TPC2) induces osteoblast differentiation and matrix mineralization while targeting autophagy

**DOI:** 10.1007/s40618-026-02898-0

**Published:** 2026-05-06

**Authors:** Azadeh Montaseri, Michela Rossi, Giulia Battafarano, Anna Riccioli, Fioretta Palombi, Claudia Giampietri, Domenico Liguoro, Rita Mancini, Martina Meucci, Biagio Palmisano, Mara Riminucci, Marco Keller, Franz Bracher, Christian Grimm, Kaoru Umehara, Wanchai De-Eknamkul, Antonio Filippini, Andrea Del Fattore

**Affiliations:** 1 Department of Anatomy, Histology, Forensic Medicine and Orthopaedics, Unit of Histology and Medical Embryology and Unit of Human Anatomy, Sapienza University of Rome, Via A. Scarpa, 16, 00161 Rome, Italy; 2 Bone Physiopathology Research Unit, Translational Pediatrics and Clinical Genetics Research Division, Bambino Gesù Children’s Hospital, IRCCS, 00146 Rome, Italy; 3 SAFU Laboratory, Department of Research, Advanced Diagnostics and Technological Innovation, Translational Research Area, IRCCS Regina Elena National Cancer Institute, 00144 Rome, Italy; 4 Department of Clinical and Molecular Medicine, Sant’Andrea Hospital, “Sapienza” University, 00185 Rome, Italy; 5 Department of Molecular Medicine, Sapienza University of Rome, 00161 Rome, Italy; 6 Department of Pharmacy – Center for Drug Research, Ludwig-Maximilians- University, Munich, Germany; 7 Immunology, Infection and Pandemic Research IIP, Fraunhofer Institute for Translational Medicine and Pharmacology ITMP, Munich, Frankfurt, Germany; 8 Walther Straub Institute of Pharmacology and Toxicology, Faculty of Medicine, Ludwig-Maximilians-University, Munich, Germany; 9 Department of Pharmacology, University of Oxford, Mansfield Rd, Oxford, OX1 3QT UK; 10 Faculty of Kampo Pharmacy, Yokohama University of Pharmacy, Matano-Cho, Totsuka-Ward, Yokohama, Kanagawa Japan; 11 Department of Pharmacognosy and Pharmaceutical Botany, Faculty of Pharmaceutical Sciences, Chulalongkorn University, Bangkok, Thailand

**Keywords:** Two-pore channel 2 (TPC2), Osteoblast, Mineralization, Autophagy, mTOR

## Abstract

**Purpose:**

Endolysosomal two-pore channels (TPCs) are non-selective cation channels that control the release of Ca^2+^ and Na^+^ from the endolysosomal lumen. TPCs also reportedly play a role in autophagy. Interestingly, autophagy regulates bone cell differentiation and function. This study aimed to provide an in-depth insight into TPC2’s action in the autophagy pathway to control osteoblast differentiation and function.

**Methods:**

Primary human mesenchymal stem cells (hMSCs) and human osteoblast-like cells (Saos-2) were used to assess osteoblastogenesis and bone mineralization, respectively. MSCs were treated with different pharmacological TPC2 inhibitors including naringenin, tetrandrine, MT-8 and SG-094 during their differentiation process. Finally, formation of osteoblasts and in vitro bone mineralization were evaluated by alkaline phosphatase, alizarin red S and Von Kossa staining. Western blot analysis was performed to investigate the expression of autophagy-related molecules.

**Results:**

The inhibition of TPC2 activity stimulates osteoblast differentiation from hMSCs and bone mineralization by Saos-2 cells. Interestingly, TPC2 inhibition reduces beclin-1 and LC3-II expression while that of the mammalian target of rapamycin (mTOR), the master regulator of autophagy, increases. Inhibition of mTOR activity by rapamycin reverses osteoblast differentiation induced by TPC2 inhibitor SG-094.

**Conclusion:**

Inhibition of TPC2 channel activity increases osteoblast differentiation and bone mineralization in vitro and interferes with the completion of autophagy, upregulating phosphorylated mTOR.

**Supplementary Information:**

The online version contains supplementary material available at 10.1007/s40618-026-02898-0.

## Introduction

The endolysosomal two-pore channels (TPCs) are localized on acidic vesicles within the endolysosomal system [1, 2]. These channels were initially identified as nicotinic acid adenine dinucleotide phosphate (NAADP)-sensitive Ca^2+^ channels [3]. In 2012, their function as PI(3,5)P_2_-gated Na^+^ channels was proposed [4]. More recently, in 2020, it was shown that TPC2 can be activated by both PI(3,5)P_2_ and NAADP, as well as their corresponding mimetics that lead to changes in cation permeability [5]. The identification of NAADP binding proteins, JPT2 and Lsm12 [6–9] revealed that NAADP activates TPCs indirectly via these auxiliary proteins. Functionally, TPC2 has been implicated in the regulation of endolysosomal trafficking as well as in autophagic processes [7, 10, 11]. The role of Ca^2+^ in autophagy appears to be context-dependent, with both stimulatory and inhibitory effects reported [12]. Accordingly, in rat astrocytes, NAADP treatment was reported to be accompanied by increased expression of beclin 1 and LC3-II [13]; this NAADP-stimulated autophagy was abolished in cells expressing a dominant-negative TPC2 mutant [13]. Conversely, TPC2 has also been proposed to act as a negative regulator of autophagy as its activation by NAADP increases lysosomal pH, which could impair autophagosome-lysosome fusion [12]. A role for autophagy in melanoma tumor stem cell biology and its interaction with TPC2 channels has also been demonstrated [14, 15]. Impaired autophagosome-lysosome fusion and increased lysosomal pH have additionally been reported in other systems, such as mouse breast cancer cells and skeletal muscle fibers [16, 17]. Interestingly, physical and functional interactions have been reported between TPC2 and mTOR, with TPC2 being inhibited by ATP via mTOR; this suggests that TPC2 senses the nutrient availability and switches to a fully open state upon nutrient deprivation [18]. Under long-term starvation, loss of TPC2 reduces mTOR activity [16]. In social amoebia Dictyostelium discoideum, TPC2 knockout modifies Ca^2+^signaling, increases lysosomal pH and mTOR levels [19]. Collectively, these findings highlight a key role for TPC2 in regulating lysosomal function and autophagic flux, both positively and negatively [12].

Osteoblasts derive from Mesenchymal stem cells (MSCs) and deposit the bone matrix through steady synthesis and secretion of macromolecules. The physiological functions of osteoblasts are finely regulated by autophagy [20]. Autophagy carefully controls osteoblast differentiation and mineralization, with compelling evidence coming from the detection of mineral needle-like crystals within autophagic vacuoles [21]. Furthermore, different autophagy-related proteins and transcription factors directly influence the biology of osteoblasts. The mechanistic/mammalian target of rapamycin (mTOR) is an evolutionary conserved serine-threonine protein kinase that is considered as the master regulator of autophagy [22]. It regulates cell growth and proliferation by sensing cellular energy status and plays a pivotal role in the control of mesenchymal stem cell differentiation into adipocytes, muscle satellite cells, osteoblasts and odontoblasts [23]. mTOR also regulates the expression of osteoblast specific transcription factors such as Runx2 (RUNT-related transcription factor 2) and osterix [24], as well as the bone mineralization [23, 24]. Overall, autophagy is considered as one of the key molecular pathways influencing osteoblast differentiation and activity.

Since bone represents the largest reservoir for ions such as Ca^2+^ and Na^+^ in the body, and TPC2 is a unique ion channel that regulates the flux of both ions, and as there is evidence that several human point mutations in TPC2 are associated with changes in bone mineral density [25], it is critical to understand if modulation of TPC2 activity affects osteoblast differentiation and function through regulation of autophagy. This study aimed to elucidate the interaction between TPC2 and the autophagy pathway in the control of osteoblast differentiation and activity, using different pharmacological inhibitors of TPC2 activity including naringenin, tetrandrine and their new, more specific and efficient derivatives i.e. MT-8 [26] and SG-094 [27]. The ability of naringenin, a flavonoid found particularly in citrus fruits, for binding directly TPC2, inhibiting its activation and abolishing the mobilization of Ca^2+^ has been established earlier [28, 29]. While naringenin only modulates TPC2 function at high concentrations, its derivative, MT-8 (pratensein), which is an O-methylated isoflavone extracted from *Dalbergia parviflora*, exhibits strong inhibitory effects on TPC2 at lower concentrations compared to naringenin [26]. Another TPC2 inhibitor used in this study is tetrandrine, a complex bisbenzylisoquinoline alkaloid from the plant *Stephania tetrandra* with the ability to block TPC2-mediated currents elicited by both NAADP and PI(3,5)P_2_ as shown previously [30]. However, as tetrandrine is toxic and non-specific for TPC2, SG-094, which is a simplified synthetic analogue of tetrandrine with enhanced efficiency of TPC2 inhibition and reduced toxicity, was developed [27]. In this study, we demonstrate that inhibition of TPC2 function enhances osteoblast formation and matrix mineralization. Furthermore, in our experimental setting, we report a blockade in the completion of the autophagy pathway; all together, these results suggest a fundamental role of TPC2 in osteoblast differentiation and function, through inhibition or dysregulation of the autophagic pathway.

## Materials and methods

### Cell culture and reagents

Human primary mesenchymal stem cells (hMSCs) of three healthy donors (biological replicates) were purchased from Lonza (PT-2501, Lonza, Switzerland). hMSCs were plated in the culture flasks containing culture medium (low glucose Dulbecco’s Modified Eagle’s Medium (DMEM)) (Euroclone, MI, Italy) supplemented with 10% FBS (Fetal Bovine Serum) (Gibco, USA), 1% penicillin/streptomycin (Sigma-Aldrich, USA) and 1% L-glutamine (Sigma-Aldrich, USA) till reaching 80–90% of confluency. To evaluate matrix mineralization, human osteosarcoma Saos-2 cell line was purchased from the American Type Culture Collection (ATCC, VA, USA). These cells were cultured and expanded in low glucose DMEM supplemented with 10% FBS, 1% penicillin/streptomycin and 1% L-glutamine.

### Osteoblastogenesis

Human primary mesenchymal stem cells were cultured in the osteogenic medium containing ascorbic acid 50 µg/ml (Sigma-Aldrich, USA) and ß-glycerophosphate 10 mM (Sigma-Aldrich, USA) for 21 days. The cells were treated with four different pharmacological TPC2 inhibitors including naringenin (Sigma-Aldrich, USA), tetrandrine (Santa Cruz Biotechnology, USA), MT-8 (isolated from *Darbergia parviflora* by Dr. Kaoru Umehara) and SG-094 (provided by Prof. Franz Bracher’s lab) at different concentrations to find the most efficient dose. As TPC2 inhibitors are dissolved in dimethyl sulfoxide (DMSO), the control cells were treated with the same amount of DMSO as vehicle. At the end of the assay the cells were fixed in paraformaldehyde (4%) and stained with alkaline phosphatase kit (86c, Sigma-Aldrich, USA) according to the manufacturer’s protocol.

### Evaluation of mineralized bone matrix

Saos-2 cells were cultured in osteogenic medium as explained before and treated with different concentrations of TPC2 inhibitors for 8 weeks. After fixation, the formation of mineralized nodules and calcium deposits were studied using alizarin Red S (ARS) (PromoCell GmbH, Germany) and Von Kossa (VK) (Bio-Optica, Italy) staining.

For ARS staining, cells were fixed in 70% cold ethanol for 1 h. According to the protocol described by da Silva Meireiles et al*.* (2008) [31], after two washes in deionized water (D/W), the cells were immersed in Alizarin Red S staining solution (2% in D/W, pH: 4.1–4.3) for 20 min at room temperature in the dark on an orbital shaker. Afterwards, the staining solution was discarded, and the cell monolayers were washed 4 times with D/W. The mineralization nodules were imaged and densitometric analysis was performed using Image J software (1.53a, NIH, USA). For von Kossa staining, cells were fixed in paraformaldehyde 4%, washed by D/W and then stained with Von Kossa staining kit reagents according to the manufacturer’s instructions. The mineralization deposits were quantified by densitometric analysis (Image J software, 1.53a, NIH, USA).

### Proliferation assay by carboxyfluorescein diacetate succinimidyl ester (CFSE) staining

Proliferation rate was evaluated incubating cells with 2 µM of CFSE (carboxyfluorescein succinimidyl ester) (ThermoFisher Scientific, USA) for 20 min at room temperature. After washing in PBS, cells were suspended in DMEM medium containing 10% FBS and were plated in 6-well dishes at a density of 1.5 × 10^5^ per well. After 24 h cells were treated with naringenin (100 µM), tetrandrine (1 µM), MT-8 (25 µM) and SG-094 (2,5 µM) for 2 days and then they were harvested by trypsin/EDTA and analyzed by BD FACSCanto^TM^II flow cytometer (BD Bioscience, USA).

### Evaluation of apoptosis by Annexin V/Propidium iodide (PI) staining

To assess the effects of TPC2 inhibitors on apoptosis, Saos-2 cells were plated at 1.5 × 10^5^ in 6-well dishes and treated with different TPC2 inhibitors for 24 h. Then, cells were trypsinized, centrifuged at 300 rcf for 7 min at 4 °C and resuspended in binding buffer containing Annexin V Pacific Blue (Thermo Fisher Scientific, IL, USA) and Propidium iodide (PI) (Sigma-Aldrich, USA) for 15 min at room temperature. The apoptosis/necrosis were analyzed by CyAn ADP flow cytometer (Beckman Coulter, CA, USA).

### Western blot analysis

At the end of the experiments, cells were washed with pre-chilled PBS and then treated with the lysis buffer containing protease and phosphatase inhibitor cocktail (Cell Signaling, Danvers, MA, USA) on ice. After sonication, the homogenized cell lysates were centrifuged at 14,000 rpm for 15 min at 4 °C and the supernatant was stored at -20 °C until further use. Total protein content of lysates was measured with Bicinchoninic acid assay (BCA) (Pierce, Rockford, IL, USA) according to the standard protocol. Samples were reduced with 2-mercaptoethanol on heat block for 5 min at 95 °C and later, the proteins were separated using SDS-PAGE under reducing conditions using 10% and 15% polyacrylamide gels and blotted onto nitrocellulose or PVDF (Amersham Bioscience, Little Chalfont, UK) membranes using a trans blot electrophoresis apparatus (Bio-Rad, USA) for 2 h at 70 V. After transferring, the membranes were incubated in the blocking buffer (5% nonfat dry milk in T-TBS) and then incubated with primary antibody overnight at 4 °C. After three times washing with T-TBS, membranes were incubated with the peroxidase-conjugated secondary antibody (Jackson Laboratories, Ann Arbor, MI, USA) for 60 min on the shaker at room temperature. Specific antigen–antibody complexes were detected by ECL reagents (Amersham, RPN2235, Italy) and visualized by the Chemidoc imaging system (Bio-Rad, USA). The intensity of protein bands was quantified by Image J (1.53a, NIH, USA) and normalized to β-actin (Cell signaling, Danvers, MA, USA). Primary antibodies used in this study are listed here: Anti-LC3 (Cell signaling, Danvers, MA, USA), Becli-1 (Cell signaling, Danvers, MA, USA), p-mTOR (Cell signaling, Danvers, MA, USA), T-mTOR (Cell signaling, USA).

### Statistical analysis

Two-tailed Student’s t-test was used for statistical comparison between the means where applicable (two groups), ordinary one-way ANOVA (for three experiments) with Dunnett’s post-test. Error bars represented mean ± SEM. All analyses were performed using Prism 8.4.2 (GraphPad Software Inc., San Diego, CA, USA).

## Results

### TPC2 inhibition stimulates osteoblast differentiation

**Fig. 1:**
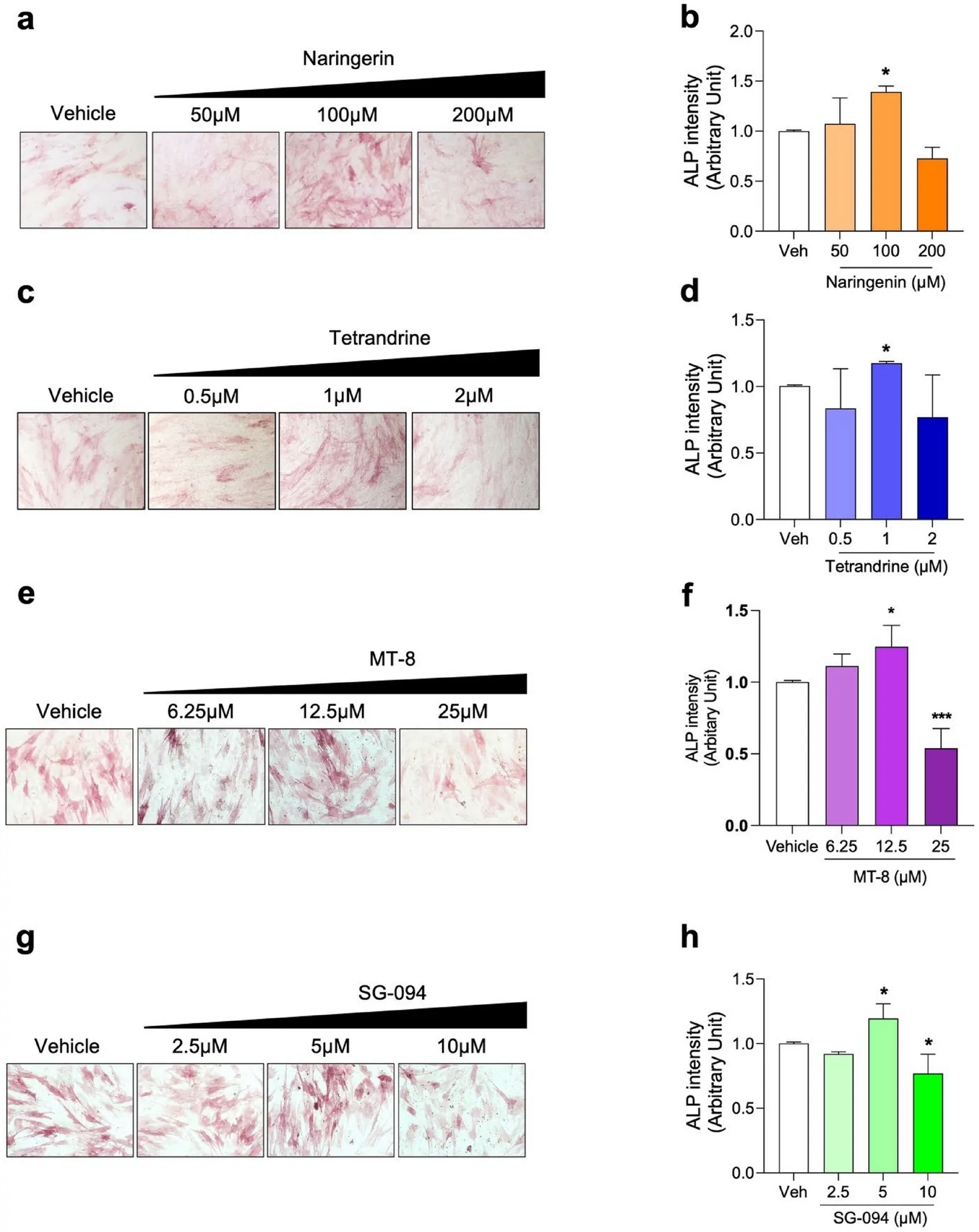

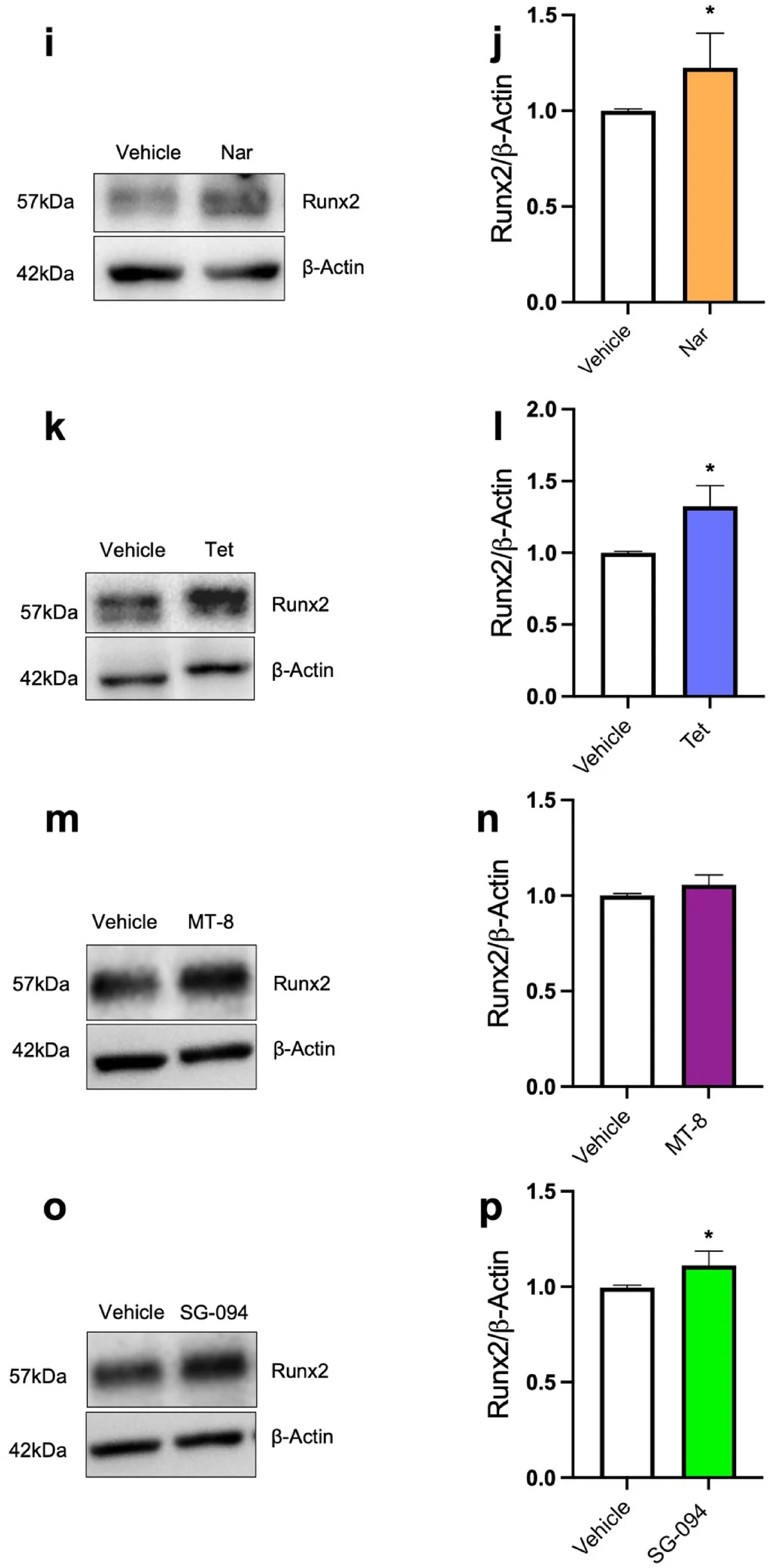
Inhibition of TPC2 channel activity induces differentiation of hMSCs into osteoblasts: Osteoblasts were differentiated from hMSC treated with osteogenic medium and different concentrations of pharmacological TPC2 inhibitors. **a, c, e, g** Representative pictures of alkaline phosphatase staining. Original magnification 10X. **b, d, f, h** Densitometric analysis of ALP staining. Expression of Runx2 by osteoblasts differentiated from hMSC and treated with TPC2 inhibitors was evaluated using western blot technique. **i, k, m, o** representative blots and (**j, l, n, p**) densitometric analysis. Results are expressed as mean ± SD of three independent biological replicates. *p ≤ 0.05, **p ≤ 0.01, ***p ≤ 0.001 *vs* vehicle-treated cells

To assess whether TPC2 inhibition alters osteoblast differentiation, hMSCs were induced to differentiate into osteoblasts in the presence of osteogenic medium supplemented with naringenin, tetrandrine, MT-8 and SG-094. Since no data are available on the effective concentrations of these pharmacological TPC2 inhibitors on osteoblast differentiation from hMSCs, cells were treated with a range of concentrations to identify the optimal conditions. As shown in Fig. 1a-h, cytochemical analysis of alkaline phosphatase (ALP) activity revealed that naringenin at 100 µM (Fig. 1a, b), tetrandrine at 1 µM (Fig. 1c, d), MT-8 at 12.5 µM (Fig. 1e, f), and SG-094 at 5 µM (Fig. 1g, h) displayed the strongest stimulatory effects on osteoblast differentiation. MT-8 at 25 µM and SG-094 at 10 µM resulted in a significant reduction in osteoblast differentiation (Fig. 1f-h). Next, MSCs were treated with selected concentrations of TPC2 inhibitors, and their differentiation was evaluated by western blot to visualize the expression of RUNT-related transcription factor 2 (Runx2) as the master transcription factor of the osteoblasts [32]. A significant increase in the expression of Runx2 was observed in osteoblasts treated with naringenin (Fig. 1i, j), tetrandrine (Fig. 1k, l) and SG-094 (Fig. 1o, p) compared to the control group.

### TPC2 inhibition impairs completion of the autophagic process in osteoblasts

**Fig. 2:**
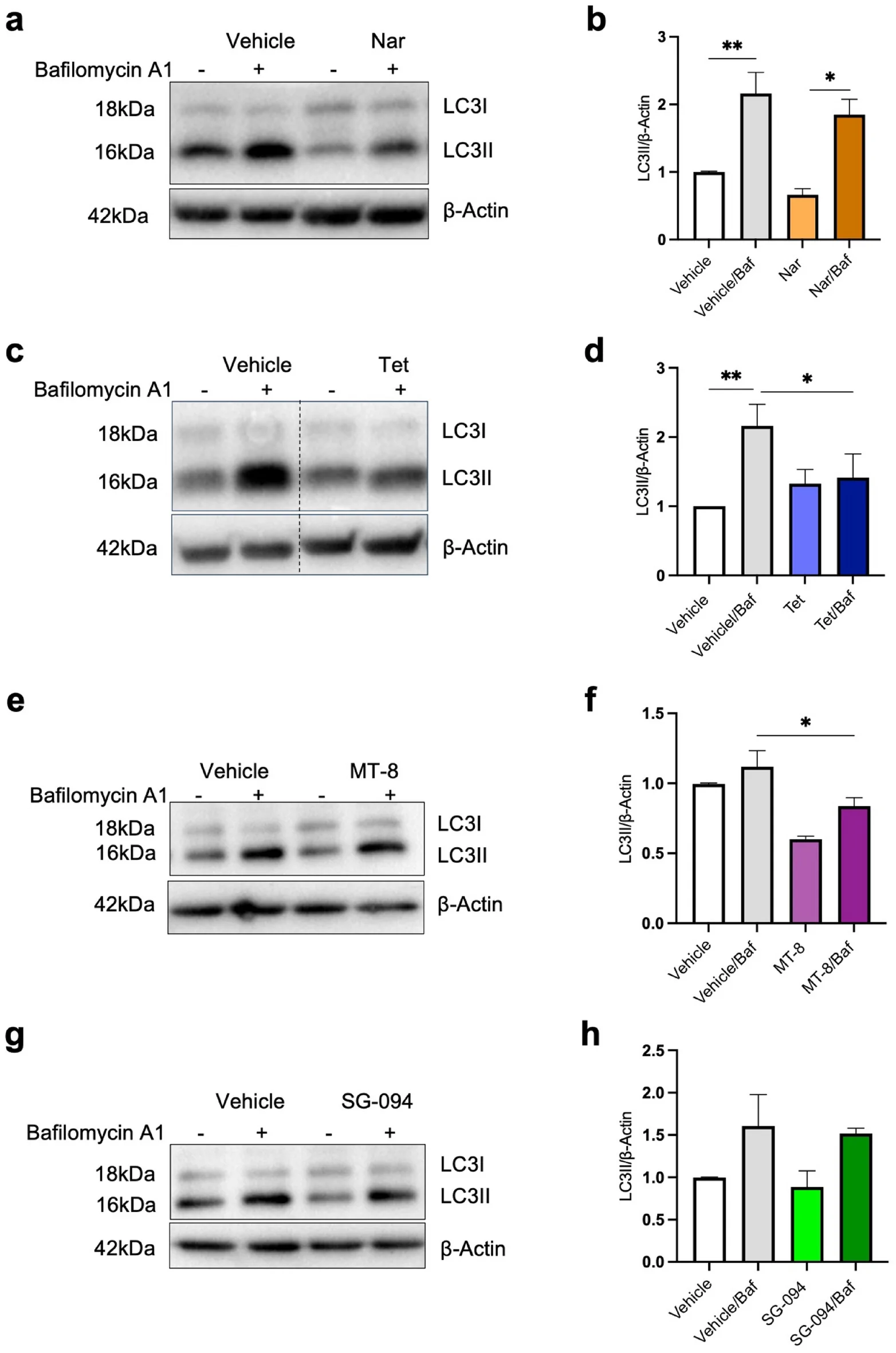

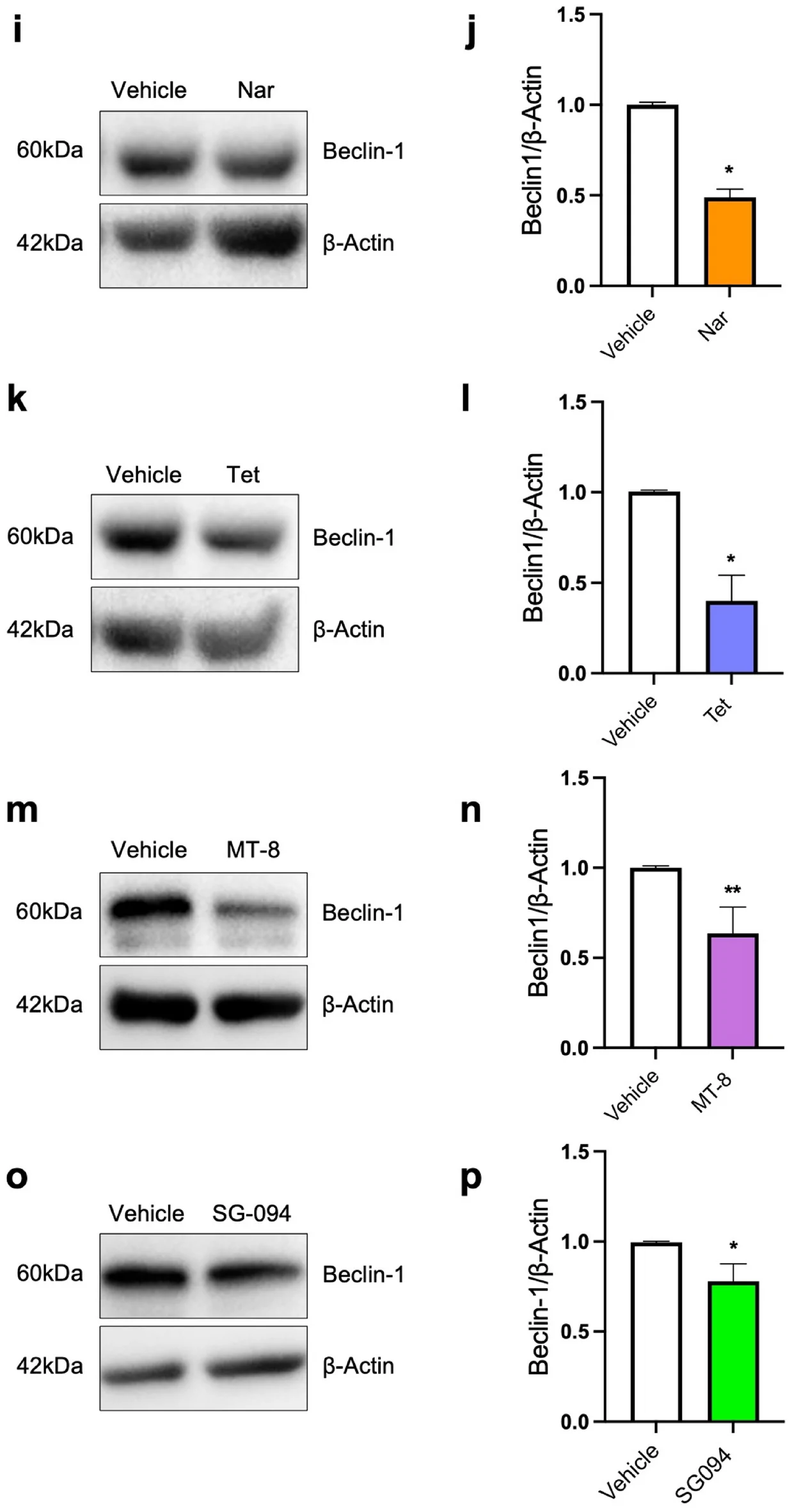
TPC2 inhibition impedes the termination of the autophagy process in osteoblasts. Western blot analysis of LC3-II expressed by osteoblasts differentiated from hMSCs and treated with naringenin (Nar) 100 µM, tetrandrine (Tet) 1 µM, MT-8 12.5 µM and SG-094 5 µM for 21 days. Three hours before termination of the experiment and being lysed, the cells were treated with bafilomycin A1. **a, c, e, g** Representative blots and (**b, d, f, h**) densitometric analysis. Furthermore, expression of Beclin-1 was evaluated in these cells: (**i, k, m, o)** representative blots and (**j, l, n, p)** densitometric analysis. Results are expressed as mean ± SD of three independent biological replicates. *p ≤ 0.05, **p ≤ 0.01 and ***p ≤ 0.001*vs* vehicle-treated cells

To explore the interaction between autophagy and TPC2 inhibition during osteoblast differentiation, expression of beclin-1 and LC3-II was evaluated in osteoblasts obtained from the differentiation of hMSCs in the presence of selected concentrations of TPC2 inhibitors. Cells were also pre-treated with Bafilomycin A1 (100 nM, for 3 h) in order to investigate whether LC3-II accumulation may result from the block of autophagosome-lysosome fusion. As shown in Fig. 2a-h, the expression of LC3-II was reduced in cells treated with tetrandrine (1 µM) and MT-8 (12.5 µM) but remained unchanged in cells treated with SG-094 (5 µM) and naringenin (100 µM) compared to vehicle-treated cells. Furthermore, inhibition of TPC2 activity caused a reduction in the expression of beclin-1 (Fig. 2i-p) These findings indicate that TPC2 inhibition could interfere with the completion of the autophagic pathway.

### Inhibition of TPC2 function upregulates the levels of phosphorylated mTOR in osteoblasts

**Fig. 3:**
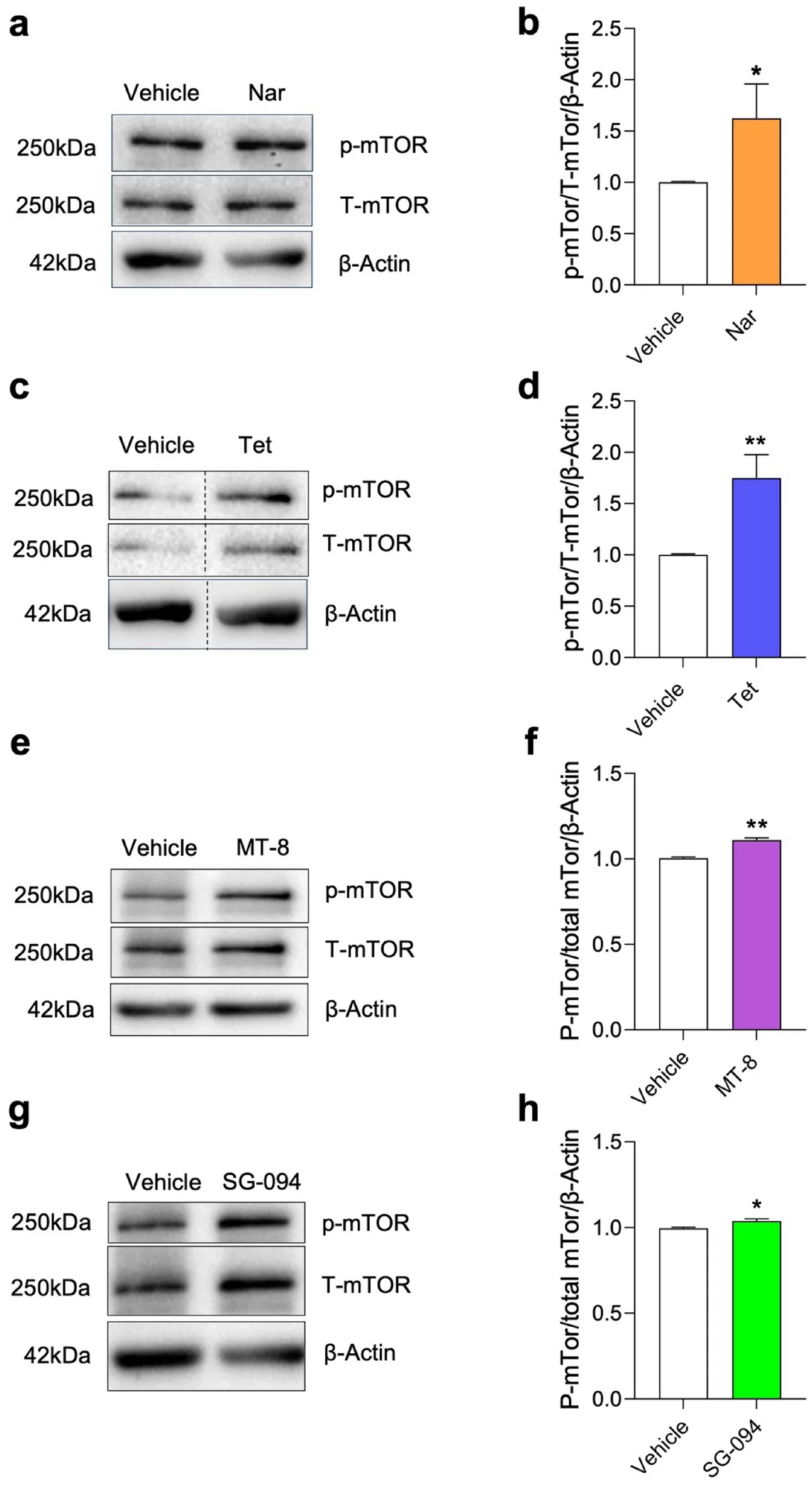
TPC2 inhibition targets mTOR expression in osteoblasts. Evaluation of (phosphorylated and total form) expression by osteoblasts using the western blot analysis. Cells were treated with Naringenin (Nar) 100 µM, Tetrandrine (Tet) 1 µM, MT-8 12.5 µM and SG-094 5 µM for 21 days. **a, c, e, g** Representative blots and (**b, d, f, h)** densitometric analysis. Results are expressed as mean ± SD of three independent biological replicates. *p ≤ 0.05 and **p ≤ 0.01 *vs* vehicle-treated cells

To further investigate the involvement of the autophagy pathway in enhanced osteoblastogenesis in the presence of TPC2 inhibitors, mTOR activation was assessed. Indeed, phosphorylated mTOR is a key negative regulator of autophagy phosphorylating principal autophagy regulators such as ULK1 and ATG13 [33]. As shown in Fig. 3a-h, treatment with TPC2 inhibitors led to an increase of the phosphorylated mTOR levels during hMSCs osteogenic differentiation, suggesting reduced autophagic activity.

### Rapamycin reverts the osteogenic effects of TPC2 inhibition

**Fig. 4:**
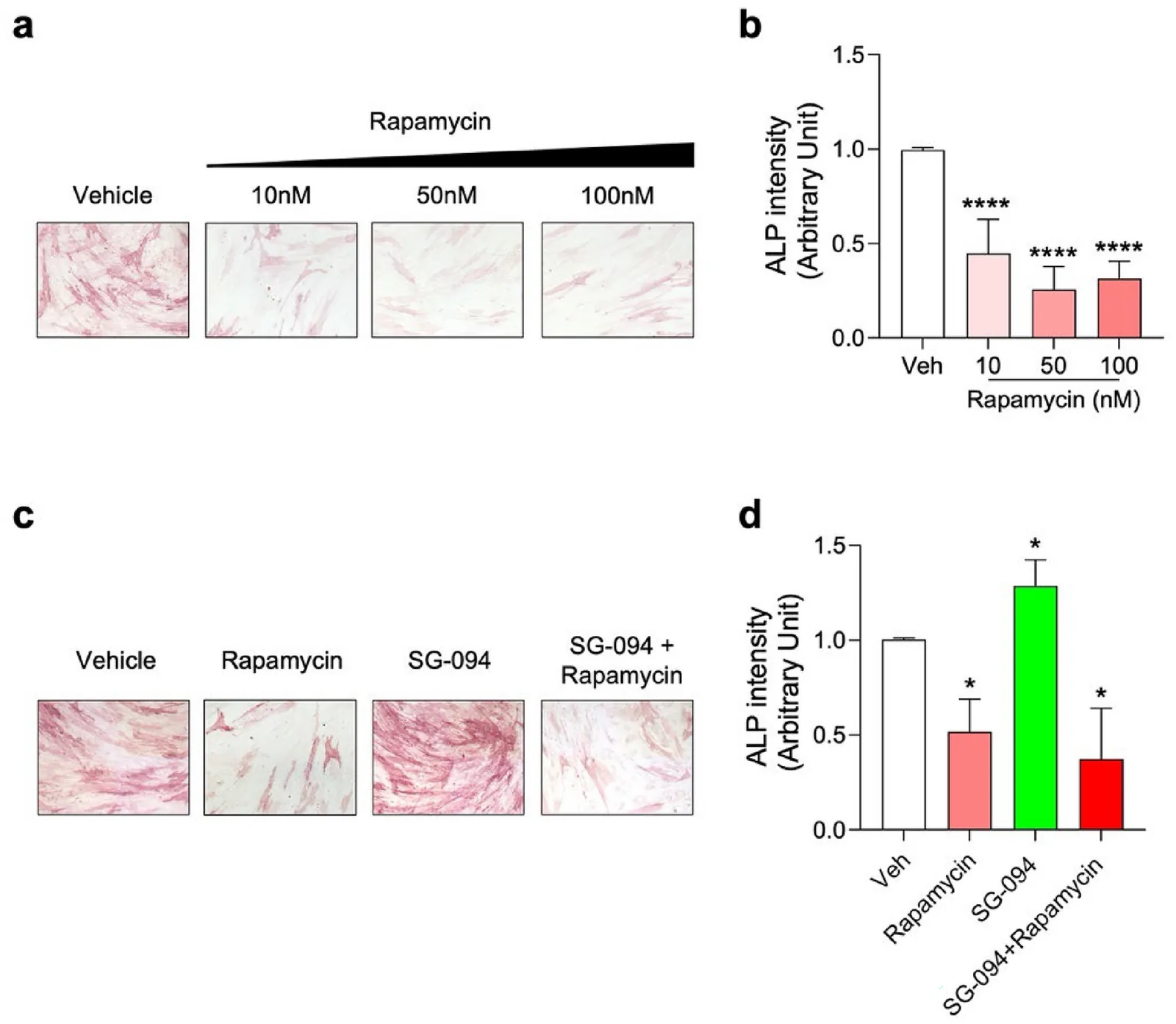
Stimulation of autophagy by Rapamycin reversed the inducing effects of SG-094 on osteoblast differentiation. **a, b** ALP staining and densitometric analysis of osteoblasts treated with DMSO + PBS as vehicle and different doses of rapamycin; (**c, d**) ALP staining and densitometric analysis of osteoblasts treated with DMSO as vehicle or with rapamycin (10 nM), SG-094 (5 µM) and combination of rapamycin and SG-094. Results are expressed as mean ± SD of three independent biological replicates. *p ≤ 0.05 and ****p ≤ 0.0001 *vs* vehicle-treated cells

To uncover the interplay between mTOR and TPC2 activity, we treated hMSCs with the mTOR inhibitor rapamycin at 10 nM, 50 nM and 100 nM for 3 weeks. All concentrations reduced osteoblastogenesis as confirmed by alkaline phosphatase staining (Fig. 4a, b). Since SG-094 is a synthetic small molecule TPC2 antagonist that leads to a closed conformation of the channel and effectively halts the ion flow through it without affecting other lysosomal cation channels [27, 34], it was selected for subsequent experiments. Combined treatment of SG-094 (5 µM) and rapamycin (10 nM) strongly reverted the stimulatory effect of SG-094 alone on osteoblast formation of hMSC (Fig. 4c, d), supporting a functional crosstalk between TPC2 and mTOR signaling during osteoblastogenesis.

### TPC2 inhibition promotes mineralization in osteoblast-like Saos-2 cells

**Fig. 5:**
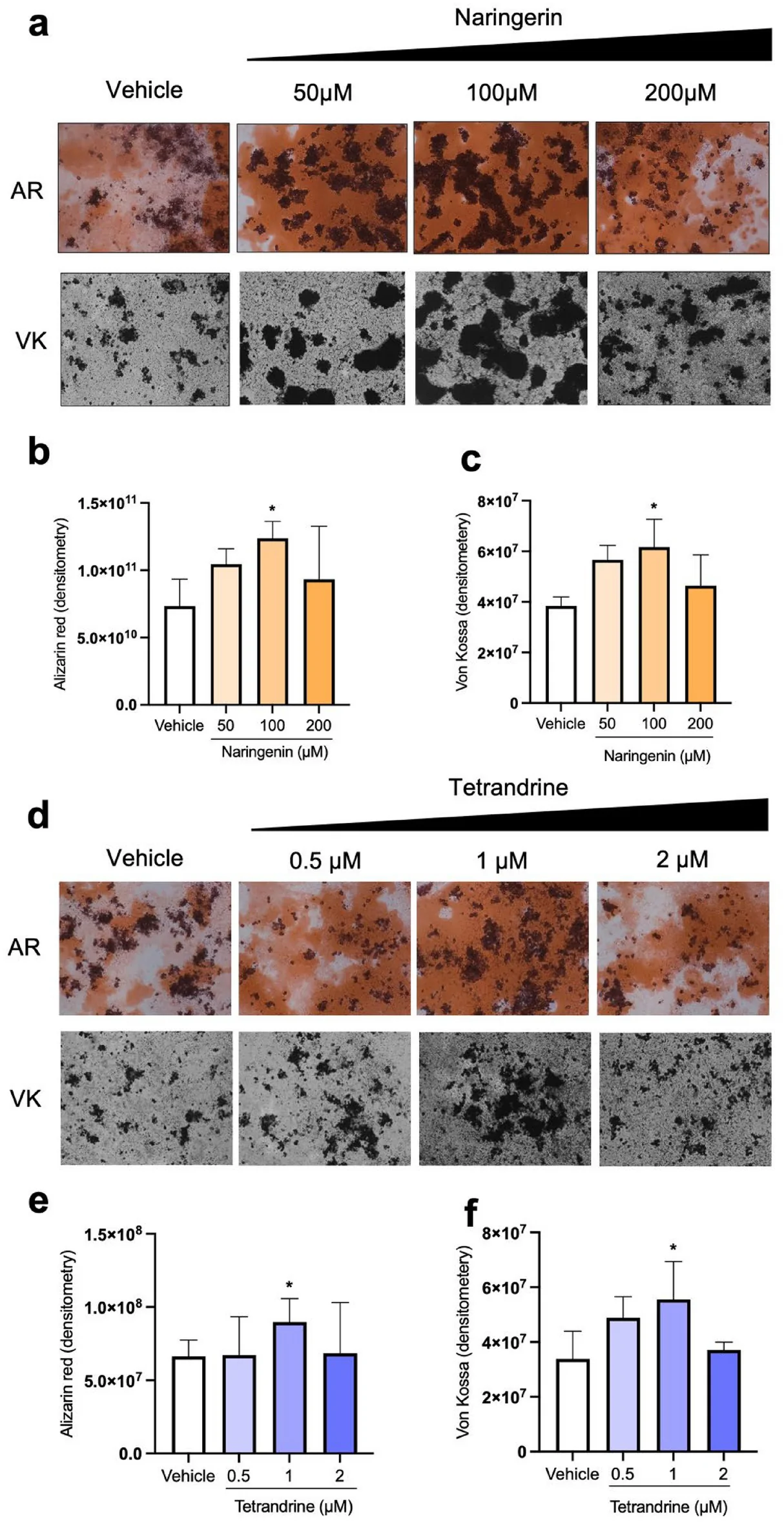

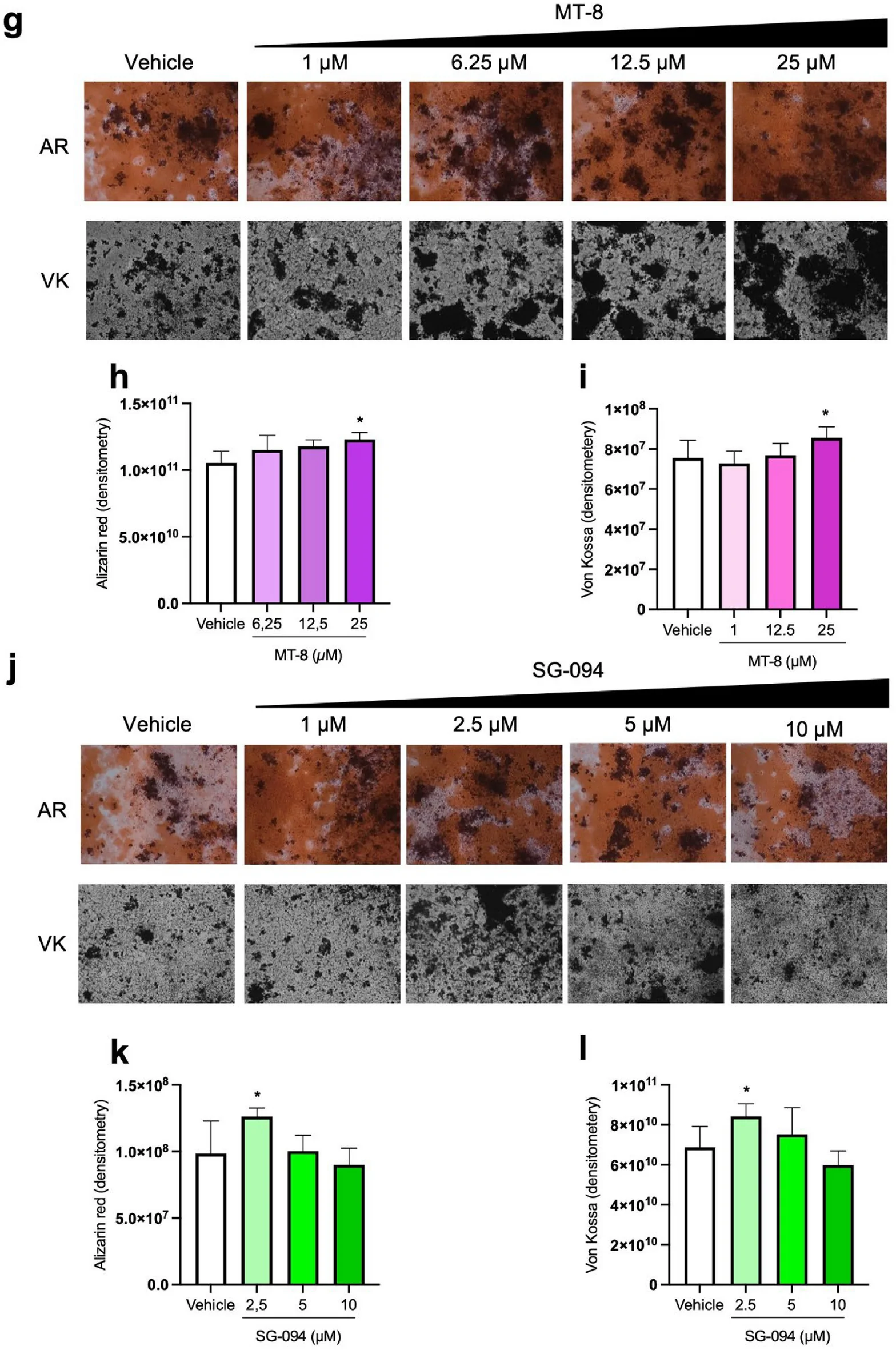
Inhibition of TPC2 activity stimulated calcium deposit formation by Saos-2 cells. Saos-2 cells treated with naringenin at different concentrations (50 µM, 100 µM and 200 µM) for 8 weeks and stained with Alizarin red S (AR) and Von Kossa (VK) staining methods. **a, d, g, j** Representative pictures of *upper panels*) Alizarin red and *lower panels*) von Kossa staining. Densitometric analysis of (**b, e, h, k**) Alizarin red and (**c, f, i, l**) Von Kossa staining. Results are expressed as mean ± SD of three independent experiments. *p ≤ 0.05 *vs* vehicle-treated cells

To explore the role of TPC2 in bone formation, Saos-2 cells were treated with TPC2 inhibitors. Alizarin Red and Von Kossa staining showed that naringenin (100 µM), tetrandrine (1 µM), MT-8 (25 µM) and SG-094 (2.5 µM) enhanced the mineralization ability (Fig. 5a-l). These doses were selected for further experiments assessing proliferation and apoptosis.

### Effects of TPC2 inhibition on proliferation and apoptosis of Saos-2 cells

**Fig. 6:**
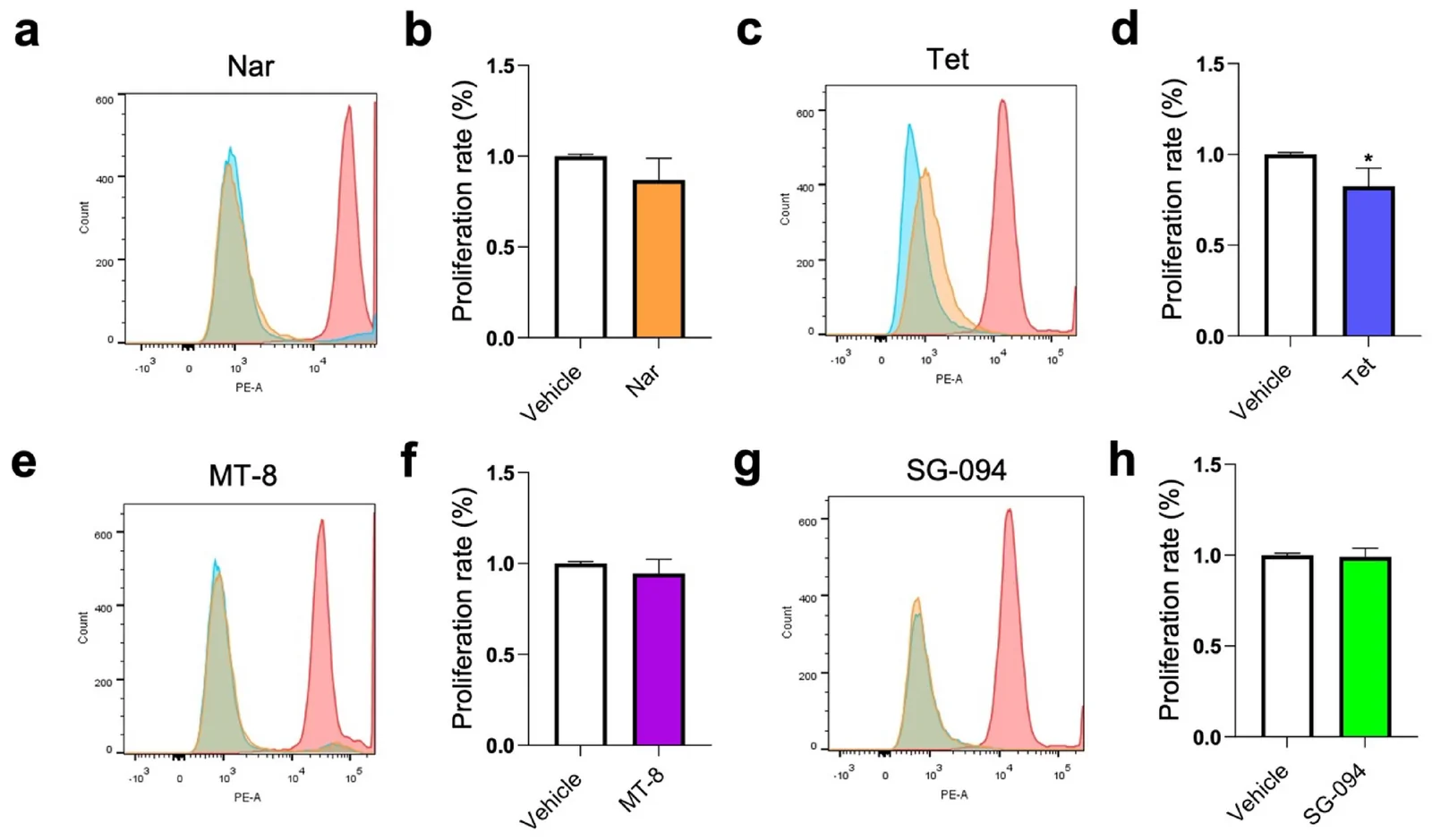
Evaluation of proliferation in Saos-2 cells after treatment by different pharmacological inhibitors of TPC2. The effects of TPC2 inhibitors on the proliferation of Saos-2 cells was evaluated using CFSE staining method. Cells were treated with the selected dose of TPC2 inhibitors for 48 h and then analysed by Flow cytometer. **a, c, e, g** Representative plots of proliferation rate; in each histogram, the pink color represents time 0, the blue color stands for vehicle and the orange one for the treated group at 48 h. **b, d, f, h** Quantification of Saos-2 cell proliferative rate after treatment with different pharmacological small molecule TPC2 inhibitors. Results are expressed as mean ± SD of four independent experiments. *p ≤ 0.05 *vs* vehicle-treated cells

Saos-2 cell proliferation was assessed using CFSE flow cytometry. No significant differences in proliferation were observed following treatment with naringenin, MT-8 and SG-094 at the applied doses while tetrandrine (1 µM) caused a slight but not significant reduction in proliferation rate (Fig. 6a-h). Moreover, none of the tested inhibitors induced apoptosis in Saos-2 cells (Supplementary Fig. 1).

### TPC2 inhibition alters autophagy in Saos-2 cells

**Fig. 7:**
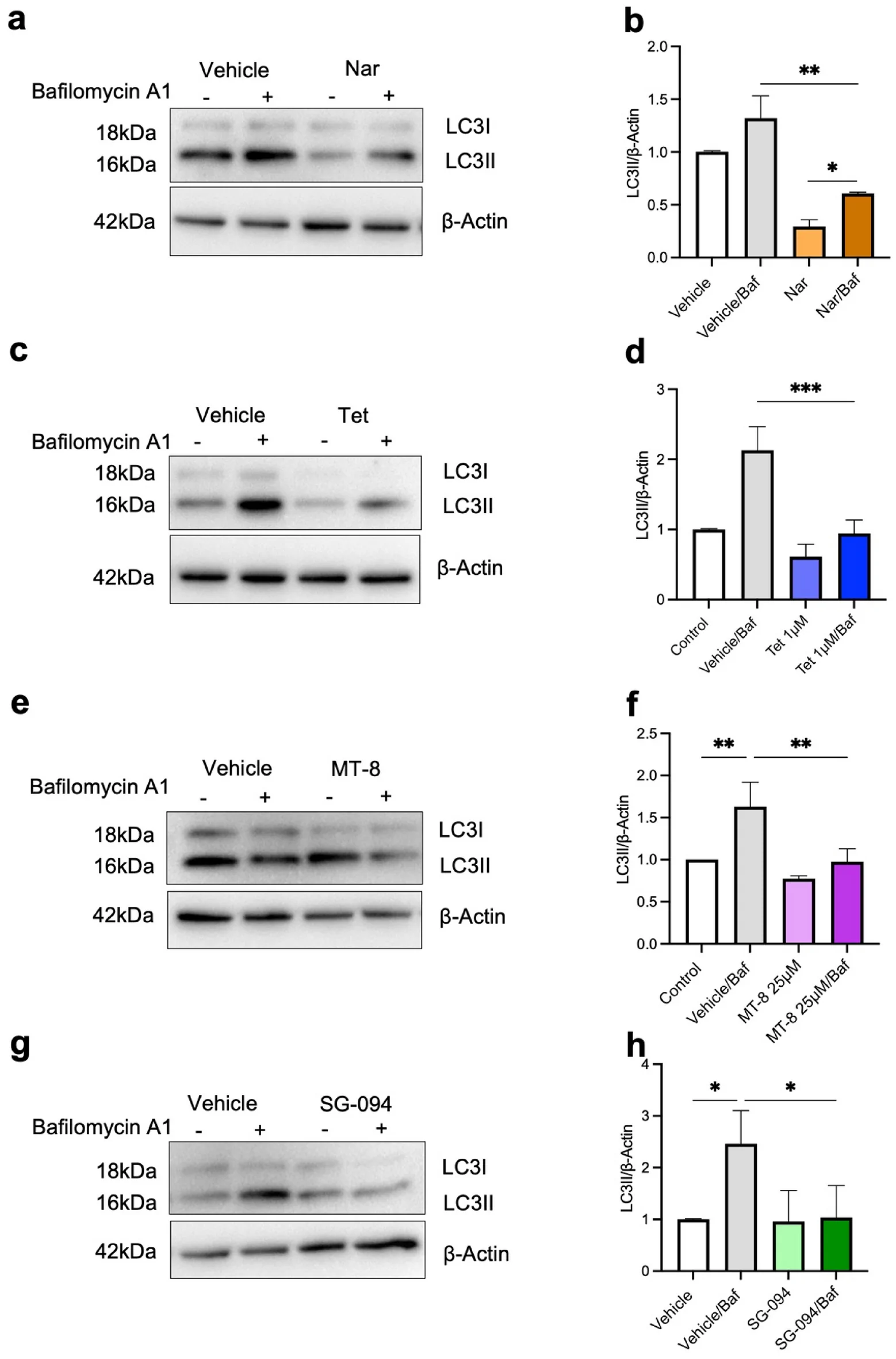

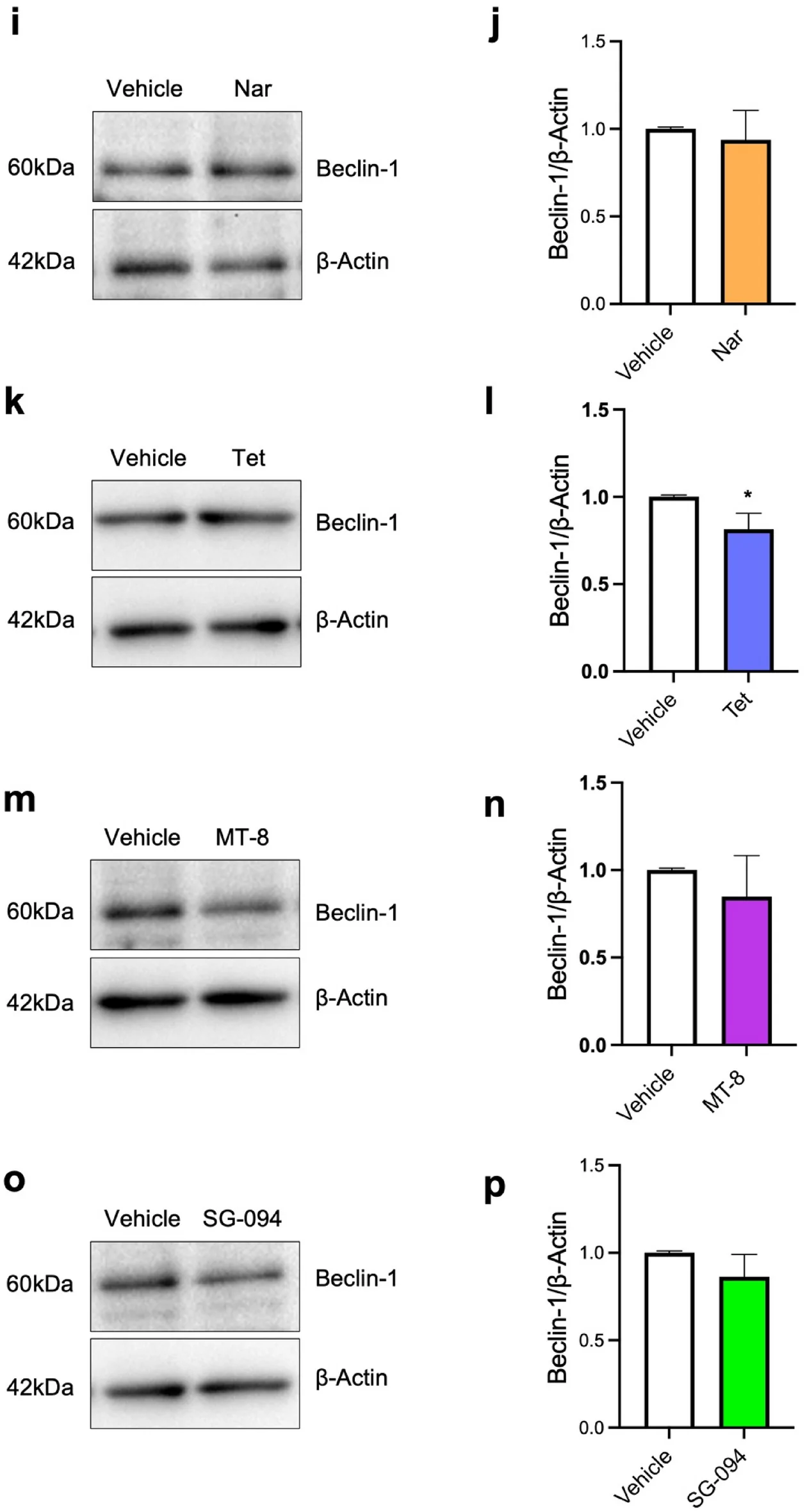
TPC2 inhibition by different pharmacological inhibitors impedes completion of the autophagy pathway. Western blot analysis was performed to measure the expression of LC3-II in Saos-2 cells treated with naringenin (Nar) 100 µM, tetrandrine (Tet) 1 µM, MT-8 12.5 µM and SG-094 5 µM. **a, c, e, g** Representative blots and (**b, d, f, h**) densitometric analysis of LC3-II expression. Expression of beclin-1 was also evaluated in Saos-2 cells treated with different TPC2 inhibitors as has demonstrated in (**i, k, m, o**) as representative blots and (**j, l, n, p**) densitometric analysis. Results are expressed as mean ± SD of three independent experiments. *p ≤ 0.05, **p ≤ 0.01 and ***p ≤ 0.001*vs* vehicle-treated cells

Finally, the relationship between TPC2 inhibition and the autophagy pathway was assessed in Saos-2 cells. Cells were treated with the selected concentrations of the TPC2 inhibitors and 3 h before being lysed were treated with Bafilomycin A1 (100 nM, for 3 h) to see if LC3-II accumulation may result from the block of autophagosome-lysosome fusion. Treatment with all four TPC2 inhibitors led to a marked reduction in LC3-II expression in Saos-2 cells compared to the vehicle cells in the presence of Bafilomycin A1 (Fig. 7a-h), indicating an inhibition of autophagic flux. Moreover, a slightly reduction in beclin-1 expression was found in Saos-2 cells treated with TPC2 inhibitors (Fig. 7i-p) that this reduction was statistically significant in cells treated with tetrandrine (Fig. 7k-l).

## Discussion

The identification of novel small molecule drugs with the ability to affect the activity of biological targets is of great importance for the treatment of bone disorders such as osteoporosis, which is a global socioeconomic burden that affects the quality of life of millions of people worldwide. In this study, testing the in vitro differentiation of hMSCs as osteogenic precursors in the presence of pharmacological TPC2 inhibitors, we uncovered that TPC2 significantly impacts human osteoblastogenesis and that autophagic flux plays a role in this process. Our data show for the first time that TPC2 inhibition enhances osteoblast differentiation from hMSCs as evidenced by increased alkaline phosphatase activity. Furthermore, we observed that inhibition of TPC2 activity promotes mineral nodule formation in human osteoblast-like Saos-2 cells.

Interestingly, LC3-II expression as a hallmark of autophagy was reduced following treatment with naringenin, tetrandrine and MT-8 and remained unchanged by SG-094 treatment in the presence of Bafilomycin A1 [34]. Its worth mentioning that TPC2 antagonist SG-094 is a synthetic analogue of tetrandrine that induces asymmetrical structural changes of the channel and stabilizes it in inactive state and is superior to other pharmacological TPC2 inhibitors because of its higher affinity and increased selectivity [34]. These observations are consistent with findings by Sun et al*.* (2018) who reported elevated LC3 levels in TPC2 overexpressing cells, whereas TPC2 silencing failed to change LC3 expression, suggesting impaired late autophagosomal-lysosomal fusion upon TPC2 modulation [17]. Also, Garcia-Rua et al*.* (2016) reported impaired autophagic flux following TPC2 silencing in starved cardiomyocytes, highlighting the key role of TPC2 in autophagy regulation [35]. Additionally, Pereira et al*.* observed reduced LC3 expression using TPC2 antagonist, *trans-*Ned-19 or by TPC2 knockdown [13, 36]. Taking together, the modified or unaltered expression of LC3-II and beclin-1 due to TPC2 inhibition suggests a block in the completion of the autophagic pathway.

In agreement with these studies, our results show that enhanced osteoblastogenesis and matrix mineralization induced by TPC2 inhibition were accompanied by disruption of autophagic flux.

We also observed increased mTOR expression in osteoblasts treated with TPC2 inhibitors, consistent with autophagy inhibition. Like TPC2, mTOR is also located to the lysosomal membrane, and both the functional and physical interaction between these two molecules have been previously reported [18]. TPC2 appears to act as a nutrient sensor, opening under low ATP conditions to regulate lysosomal pH and amino acid homeostasis [18]. Cang et al. (2013) and Chao et al*.* (2017) demonstrated that mTOR inhibition (via rapamycin and torin-1) increases TPC2 activity, confirming a link between these two molecules [18, 37]. Collectively, these studies suggest that TPC2 and mTOR act within the same signaling complex to regulate autophagy and cellular energy homeostasis [16].

Importantly, we found that rapamycin inhibits osteoblastogenesis and reduces alkaline phosphatase activity in hMSC-derived cells co-treated with SG-094, further supporting a functional interaction between mTOR and TPC2 in osteoblast differentiation.

These findings are consistent with reports that mTOR regulates stem cell differentiation and mineralization [38]. For example, Kim et al*.* (2011) demonstrated that mTOR silencing or inhibition in human dental pulp stem cells suppresses extracellular matrix molecules expression (e.g., sialoproteins) and mineral deposition. [39]. Takeuchi et al*.* (2019) further corroborated the involvement of Runx-2/mTOR pathway in odontoblast differentiation and matrix mineralization, immunolocalizing both proteins in newly differentiated odontoblast-like cells [38].

## Conclusion

**Fig. 8:**
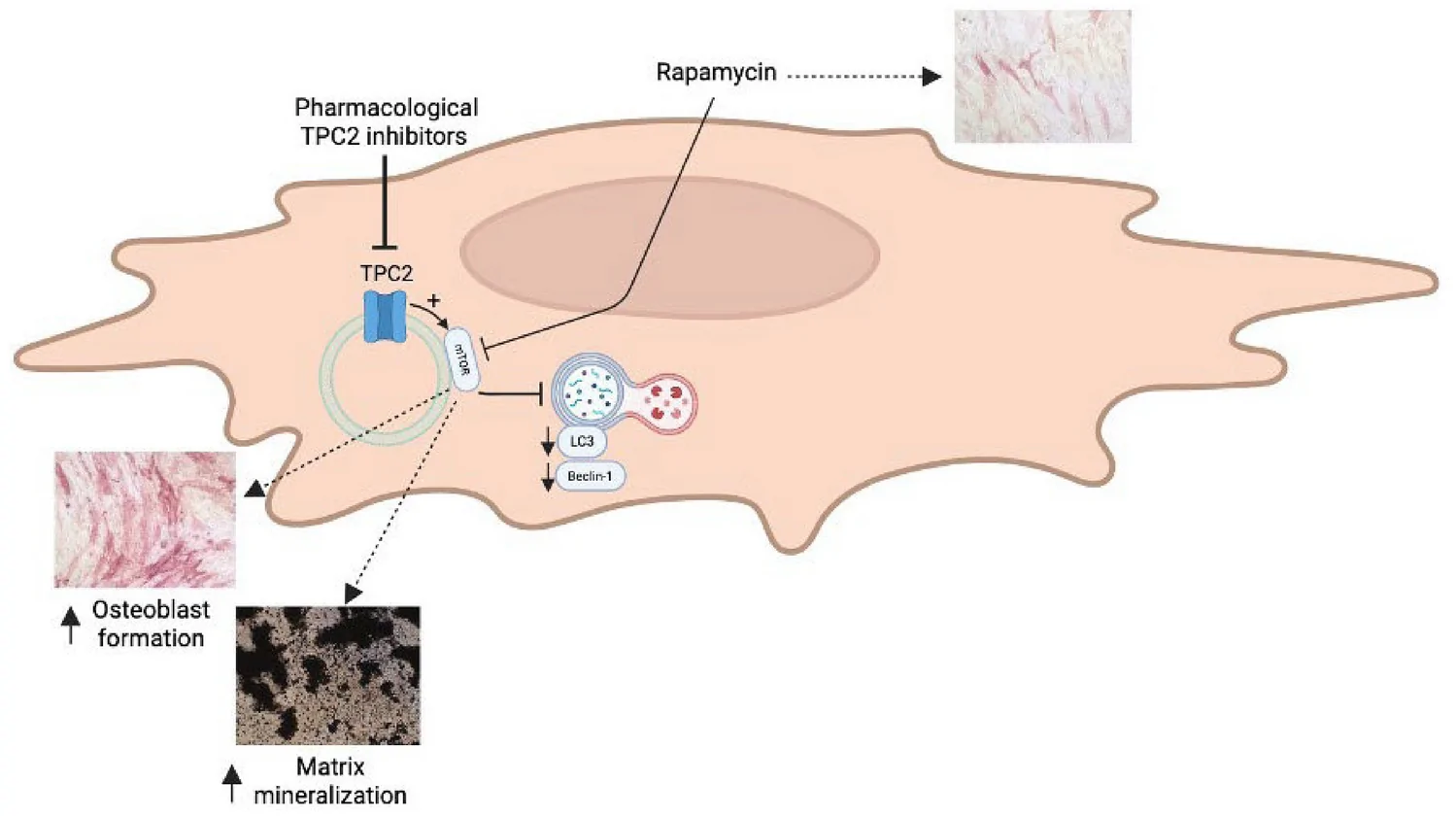
Schematic representation of the TPC2 inhibitors activity on osteoblast formation and function via autophagy

In conclusion, our study provides new evidence for the role of TPC2 in osteoblast differentiation and function, linking its inhibition to impaired autophagic flux and increased mTOR activity. Pharmacological inhibition of TPC2 activity enhanced osteoblastogenesis and stimulated matrix calcification, effects that were reversed by mTOR inhibition with rapamycin (Fig. 8). Within the limitations imposed by our experimental model *i.e.* long-term culture of hMSCs and Saos-2 cells, our study highlights a potential therapeutic strategy to be further tested preclinically and potentially in clinical applications for the targeted delivery of TPC2 inhibitors to bone tissue using different methods such as functionalized nanoparticles to treat conditions characterized by reduced bone mineral density. Prospectively*, *in vivo experiments may pave the roads for new possible therapeutic approaches.

## Supplementary Information

Below is the link to the electronic supplementary material.

Supplementary file1

## Data Availability

The data that support the findings of this study are available from the corresponding author upon request. All data generated or analyzed during this study are included in this published article.
