# Supplementary material for: Inhibition of endolysosomal two-pore channel 2 (TPC2) induces osteoblast differentiation and matrix mineralization while targeting autophagy
Source: J Endocrinol Invest. 2026 May 6;49(9):2421–38. doi: 10.1007/s40618-026-02898-0 (PMC13498500; doi:10.1007/s40618-026-02898-0)
Supplement: Supplementary file 1 [file 40618_2026_2898_MOESM1_ESM.docx]

**Supplementary Fig. 1 Evaluation of apoptosis in Saos-2 cells treated with different pharmacological small molecule TPC2 inhibitors.** To study the effects of TPC2 inhibitors on apoptosis in osteoblasts, Saos-2 cells were treated with the selected dose of each TPC2 inhibitor for 48 hours. Cells were stained with Annexin V/Propidium iodide (PI) and the apoptotic rate was analysed by Flow cytometer. **a, c, e, g)** Representative plots of Annexin/PI staining and **b, d, f, h)** quantification of early and late apoptotic cell populations stained with annexin V/PI in vehicle and TPC2 inhibitor-treated cells. Results are expressed as mean±SD of four independent experiments
